# Fishing behavior in the red fox: Opportunistic‐caching behavior or surplus killing?

**DOI:** 10.1002/ecy.3814

**Published:** 2022-08-18

**Authors:** Jorge Tobajas, Francisco Díaz‐Ruiz

**Affiliations:** ^1^ Departamento de Botánica, Ecología y Fisiología Vegetal Universidad de Córdoba Córdoba Spain; ^2^ Instituto de Investigación en Recursos Cinegéticos (IREC) CSIC‐UCLM‐JCCM Ciudad Real Spain; ^3^ Biodiversity Research Institute (CSIC ‐ Oviedo University ‐ Principality of Asturias) University of Oviedo Mieres Spain; ^4^ Biogeography, Diversity, and Conservation Research Team, Dept. Biología Animal, Facultad de Ciencias Universidad de Málaga Málaga Spain

**Keywords:** behavioral ecology, canids, *Cyprinus carpio*, diet, feeding behavior, fish, mammalian predators, predation, *Vulpes vulpes*

The red fox (*Vulpes vulpes*) is a mesocarnivore species that exploits opportunistically a wide range of prey items that are consumed as a function of their abundance and availability (Díaz‐Ruiz et al., 2013). Fish are an unusual prey group in the diet of red foxes that is occasionally reported in dietary studies (e.g., Basuony et al., 2005; Wagnon & Serfass, 2017), but we do not know if the red fox obtains fish from scavenging or active hunting. Here, we report what may be the first known case of several fish hunted by a red fox. We raise different reasons to explain the observed novel behavior and discuss its ecological implications.

While doing field work in the surroundings of the Valuengo reservoir in southern Extremadura (Spain; 38.294845° N, −6.674353° W), we observed an adult male red fox catching European carps (*Cyprinus carpio*) on the shore (Figure 1 and Videos [Link], [Link]). The observation took place on 24 March 2016, between 1:18 and 2:51 PM. During this time period, until it noticed our presence and left the area, the individual hunted 10 medium‐sized carps (~1 kg) out of 12 observed attempts, a capture success rate of 83%. The male fox approached the water's edge, where the carps spawned their eggs and while they were distracted by the frenzy of their reproduction (Figure 1 and Video S5), and jumped into the water to catch the fish (Figure 1 and Videos [Link], [Link]). After each capture, the fox moved away ~20–30 m from the shore to leave, hide, or bury the captured prey that could be understood as a potential caching behavior, presumably for later consumption (Video S3). On one occasion, the fox took one fish and left the area and moved to a scrubby area further away. The observation distance was ~100 m with binoculars and recorded by a reflex camera with a telephoto lens. This allowed us to observe how the fox never consumed any substantial part of the captured prey. However, on several occasions we observed that the fox seemed to eat small parts of the prey (e.g., Videos S2 and S3). These parts might be some eggs from the pregnant female carps, but the lack of direct vision prevented us to unequivocally confirm this activity. After 52 min, a female fox (easily identifiable by docked tail) appeared from the same area, took a large carp that the male had hunted and carried it toward the scrubland (Video S4) without any interference from the male. The purpose of these captures may have been to supply prey for the female and the pups, because the observations occurred during the known breeding season of red foxes in Iberian Mediterranean habitats (Zapata et al., 1997). However, we did not observe any pups, so this assumption remains unconfirmed.

**FIGURE 1:**
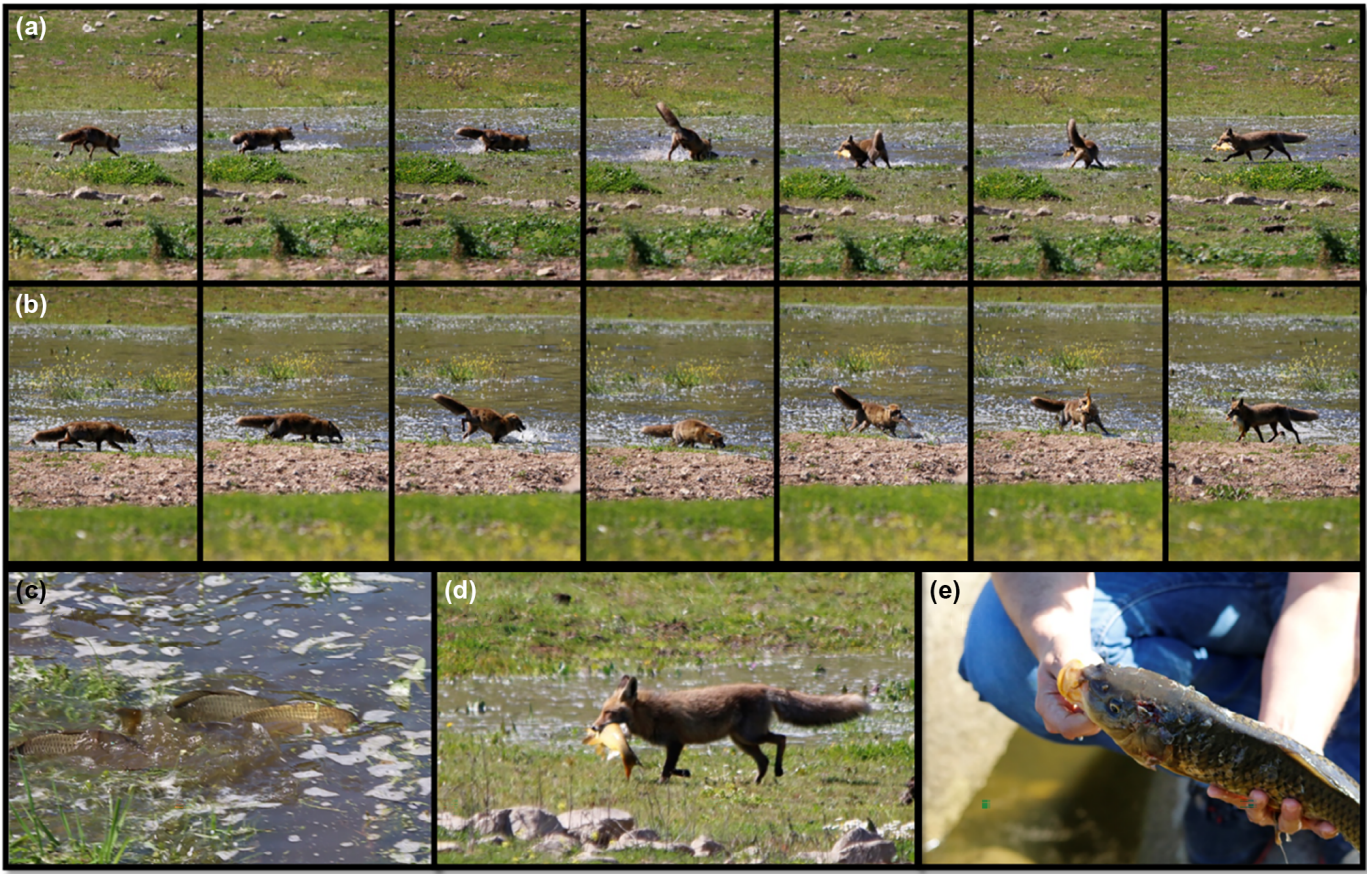
(a, b) Two sequences showing the red fox (*Vulpes vulpes*) hunting European carps (*Cyprinus carpio*) on the shore of the Valuengo reservoir (southern Extremadura; Spain) during the carp spawning period, March 2016. (c) European carps spawning eggs and distracted by the frenzy of reproduction in the shallow reservoir shore. (d) Red fox carrying a large European carp far to the shore. (e) European carp hunted by the red fox with the fatal incisions on the head.

Our observation has several implications for canid behavioral ecology. First, this observation shows the ability of this species to catch fish in their environment and confirms fish as a food item that can be consumed as a fresh capture and not only opportunistically as carrion (Basuony et al., 2005). This behavior has been described previously for gray wolves (*Canis lupus*) in the USA (e.g., Darimont et al., 2003; Gable et al., 2018), but not documented for red foxes. But the most novel aspect of the observation was the fact that large numbers of fish were caught in such a short period of time and with a high effectiveness, documenting (photographically and by video) for the first time this hunting behavior. This behavior may not be an isolated behavior associated with a single individual, and probably other individuals can do this as well. This could imply that this opportunistic behavior is an intrinsic characteristic of foxes as consequence of ephemeral high‐prey availability, similar to that described for gray wolves and salmon in British Columbia and Alaska (Darimont et al., 2003; Stanek et al., 2017). Notwithstanding, perhaps not all red fox individuals are able to develop this hunting behavior so efficiently (capture success rate >80%) suggesting that this could be a learned behavior based on previous experience (Darimont et al., 2003; Stanek et al., 2017).

At first glance, our observation might fit with a case of “surplus killing”, because the red fox invested large efforts in killing a large number of fish that it apparently did not immediately consume (Kruuk, 1972). The observation took place during carp spawning, which in reservoirs occurs in large groups on shallow banks (Doadrio, 2002), making them more vulnerable to predation. The fact that it occurred at the moment of maximum availability and vulnerability of the prey concurs with previous hypotheses about the triggers of surplus killing (please refer to Kruuk, 1972; Lincoln & Quinn, 2019; Short et al., 2002; Wiesel, 2010). However, in some cases apparent surplus killing may be consistent with the optimal foraging theory, and may be considered as an adaptive behavior. For instance, this occurs when one or more individuals kill a large number of prey that they do not consume but are exploited by conspecifics of the same social unit (Kruuk, 1972) or when there is a selective consumption of optimal carcass discarding low quality ones (Darimont et al., 2003; Lincoln & Quinn, 2019). The fact that the female, most likely breeding (Zapata et al., 1997), and most likely to be a partner of the fox that hunted the fish, took part of the catch without the male preventing it (Video S4), indicates that the male was capturing and caching prey to feed the family group (i.e., female and pups) that at that time was highly dependent on the male (Macdonald, 1979). Therefore, it could be understood that there is a use for and optimization of the resource, at least partially, so that a behavior could be occurring with the aim of obtaining a large amount of necessary resources with little effort, which would fall within the optimal foraging theory (Stephens & Krebs, 1986). A similar behavior has been described for arctic foxes (*Vulpes lagopus*) that cache large amounts of bird eggs during the reproduction season to feed their pups (Clermont et al., 2021; Giroux et al., 2012). Additionally, the male fox seemed to feed on some eggs from the female carps (e.g., Videos S2 and S3), which would be consistent with a selective consumption of the carcasses and discarding lower quality tissues, behavior previously described for brown bears (*Ursus arctos*) and gray wolves under high availability of salmon in British Columbia and Alaska (Darimont et al., 2003; Lincoln & Quinn, 2019). Accordingly, we hypothesize that the observed behavior follows the optimal foraging theory, giving less support to surplus killing.

It is of key importance that these types of field observations be reported and published because these behavioral phenomena are very difficult to observe in the wild. Furthermore, the uncommon nature of these behaviors makes it difficult to test hypotheses in nature using designed experiments. Therefore, the accumulation of this kind of evidence will contribute to the development of more robust studies that will lead to a better understanding of these behaviors and their ecological implications (Lincoln & Quinn, 2019).

## CONFLICT OF INTEREST

Authors declare no conflict of interests.

## Supporting information


Video S1

Video S2

Video S3

Video S4

Video S5

Video S1 Legend

Video S2 Legend

Video S3 Legend

Video S4 Legend

Video S5 Legend
