# Supplementary material for: Fishing behavior in the red fox: Opportunistic‐caching behavior or surplus killing?
Source: Ecology. 2022 Aug 18;103(12):e3814. doi: 10.1002/ecy.3814 (PMC10078576; doi:10.1002/ecy.3814)
Supplement: Supplementary file 6 — Video S1 Legend [file ECY-103-0-s004.pdf]

Supporting Information. Jorge Tobajas and Francisco Díaz-Ruiz. Fishing behavior in the red fox: Opportunistic-caching behavior or surplus killing? *Ecology*.

Video S1. Fishing behavior of a male red fox (*Vulpes vulpes*). The red fox hunt actively a European Carp (*Cyprinus carpio*) in the shore of the Valuengo reservoir in southern Extremadura (Spain; 38.294845 N, -6.674353 W). The male fox approached the water's edge, where cyprinids spawned their eggs and while they were distracted by the frenzy of reproduction, and jumped into the water to catch the fishes. Author Jorge Tobajas.
