# Supplementary material for: Fishing behavior in the red fox: Opportunistic‐caching behavior or surplus killing?
Source: Ecology. 2022 Aug 18;103(12):e3814. doi: 10.1002/ecy.3814 (PMC10078576; doi:10.1002/ecy.3814)
Supplement: Supplementary file 8 — Video S3 Legend [file ECY-103-0-s003.pdf]

Supporting Information. Jorge Tobajas and Francisco Díaz-Ruiz. Fishing behavior in the red fox: Opportunistic-caching behavior or surplus killing? *Ecology.*

Video S4. Cooperative behavior of a couple of red foxes (*Vulpes vulpes*). After 52 min, a female fox (easily identifiable by docked tail) appeared from the same area, took a large European carp (*Cyprinus carpio*) that the male had hunted and carried it towards the scrubland without any interference from the male. The video was recorded in the shore of the Valuengo reservoir in southern Extremadura (Spain; 38.294845 N, -6.674353 W). Author Jorge Tobajas.
