# Supplementary material for: Fishing behavior in the red fox: Opportunistic‐caching behavior or surplus killing?
Source: Ecology. 2022 Aug 18;103(12):e3814. doi: 10.1002/ecy.3814 (PMC10078576; doi:10.1002/ecy.3814)
Supplement: Supplementary file 9 — Video S4 Legend [file ECY-103-0-s007.pdf]

Supporting Information. Jorge Tobajas and Francisco Díaz-Ruiz. Fishing behavior in the red fox: Opportunistic-caching behavior or surplus killing? *Ecology.*

Video S3. Fishing behavior of a male red fox (*Vulpes vulpes*). After hiding with sand a recently hunted European Carp (*Cyprinus carpio*) in the shore of the Valuengo reservoir in southern Extremadura (Spain; 38.294845 N, -6.674353 W), the male fox immediately go to catch another European Carp. Author Jorge Tobajas.
